# Anthropometric determinants of lung function in apparently healthy individuals

**DOI:** 10.4102/sajp.v77i1.1509

**Published:** 2021-01-15

**Authors:** Michael O. Ogunlana, Olufemi O. Oyewole, Adetutu I. Lateef, Ayomikun F. Ayodeji

**Affiliations:** 1 Department of Physiotherapy, Federal Medical Centre Abeokuta, Abeokuta, Nigeria; 2 Department of Health Sciences, University of KwaZulu Natal, Durban, South Africa; 3 Department of Physiotherapy, Olabisi Onabanjo University Teaching Hospital, Sagamu, Nigeria

## Abstract

**Background:**

Forced vital capacity (FVC) and peak expiratory flow rate (PEFR) are used to assess and monitor the management of lung pathology.

**Objectives:**

Our study documented spirometry reference values for apparently healthy Nigerians and developed predictive equations for pulmonary function.

**Method:**

A cross-sectional survey involving healthy adult Nigerians included anthropometric measurements of weight, height, waist, hip circumference (HC), sagittal abdominal diameter (SAD) and percentage body fat. Anthropometric indices (body mass index [BMI] and waist-to-hip ratio [WHR]) were estimated and pulmonary function tests (FVC, forced expiratory volume in 1 s [FEV1], PEFR, FEV1/FVC ratio) measured. The association amongst selected anthropometric and socio-demographic variables and pulmonary function test parameters were established using *t*-tests and Pearson’s product moment correlation tests. The predictors of pulmonary function were established using stepwise multiple linear regression models.

**Results:**

Four hundred and forty-four adults (156 [35.1%] men) were included, mean age 37.3 ± 8.25 (range 22–25) years. Male participants had significantly higher lung volumes than females (*p* < 0.05). Age, height, weight and percentage body fat had significant low correlations with lung function test parameters (*p* < 0.05). Fat-free mass (FFM), fat mass (FM), SAD, height and age of participants were main predictors of FVC and FEV1 (*R*^2^ = 0.43 and 0.41, respectively). Fat-free mass and SAD were main predictors of PEFR (*R*^2^ = 0.53). Sagittal abdominal diameter and age were main predictors of FEV1/FVC ratio (*R*^2^ = 0.34).

**Conclusion:**

Fat-free mass, FM, height, age and SAD are important determinants of lung volumes and key variables for predictive equations of pulmonary function.

**Clinical implications:**

An accurate documentation of pulmonary function values for apparently healthy Nigerian adults may be useful in identifying deviations from normative values thereby giving an index of suspicion for the diagnosis of pulmonary dysfunction.

**Keywords:**

anthropometric; lung function; spirometry; fat-free mass; apparently healthy.

## Introduction

The associations between human body dimensions (in composition and fat distribution) and the functionality of the human lungs for gaseous exchange have been extensively documented (Lazarus, Sparrow & Weiss 1997; Oloyede, Ekrikpo & Ekanem 2013; Santana et al. 2001; Soundariya & Neelambikai 2013; Wannamethee et al. 2005). Intrinsic non-modifiable factors such as age and sex influence the functionality of the pulmonary system (Caussade 2008; Ne’ve et al. 2002). Body composition changes with age, with increases in fat mass (FM) and visceral fat and declining skeletal muscle mass, whilst lung function also declines with age (Santana et al. 2001). Male subjects are often purported to have better lung function than females because of the preponderance of FM in the latter (Bajo & Mangued 2010). Hence, the influence of age and sex on the functionality of human lungs for gaseous exchange can be explained by anthropometric proportionalities.

Anthropometry deals with the measurement of the size, weight and proportions of the human body (Atha & Andrew 2014). It also includes body composition measurement that involves assessment of body fat distribution with an emphasis on total body fat, fat-free mass (FFM) and percentage body fat. Anthropometric measurements use fat distribution indexes such as body mass index (BMI), waist-to-hip ratio (WHR), skinfold and sagittal abdominal diameter (SAD) measurements to describe body fat distribution.

Lung function tests have become an indispensable tool for clinical evaluation of respiratory health and disease (Liang, Lam & Feng 2012). Spirometry is a relatively simple, non-invasive method for measuring the volume of air in the lung at maximal inhalation as a function of time using forced manoeuvres (Ferguson et al. 2000). It is widely accepted as a clinical tool for diagnosing obstructive, restrictive or mixed ventilatory defects such as chronic obstructive pulmonary diseases, interstitial lung diseases and asthma (Pellegrino et al. 2005). Objective measurements of pulmonary function can be useful in the diagnostic evaluation of patients who have a cough, exercise limitation or other symptoms and signs attributed to the respiratory system.

Spirometry manoeuvres, instruments or technologies and predictive equations may vary with the study populations (Enright, Skloot & Herbert 2008). It is important to develop population-specific spirometric prediction equations to ensure the reliability of lung function evaluation (Nysom et al. 1997). This may be equally important in developing countries where spirometers are not easily accessible. In Nigeria, such predictive equations are not well documented and the lung function of healthy Nigerians is not well characterised. Our study therefore aimed to document spirometry reference values for apparently healthy Nigerians and to develop predictive equations for pulmonary function parameters using anthropometric variables and indexes. The association amongst different anthropometric parameters and lung function test variables was also investigated.

## Methods

This cross-sectional survey included participants who were healthy civil servants working at two Government agencies and one private office environment in Abeokuta Ogun State, Nigeria.

Participants with known medical illness or a history of drug use were excluded. Written informed consent was obtained from all participants. A detailed review of their medical history through a structured questionnaire and physical examination was obtained. The participants were instructed to wear lightweight clothing during their study visit.

During the anthropometric measurement, participants were instructed to stand erect with the abdomen relaxed, arms at their sides and feet together. Weight (kg) was recorded using a standard weighing scale. Height (m) was measured in standing in an erect posture using a stadiometer and the BMI was calculated. Waist circumference (WC) (cm) was measured at the narrowest circumference between the bottom of the rib cage and the top of the iliac crest after normal respiration. Hip circumference (HC) (cm) was measured at the maximum circumference over the buttocks. Waist-to-hip ratio was calculated. The SAD was measured with a portable, sliding beam, abdominal caliper whilst the participants were in supine on a flat standard hospital bed. The SAD was measured as the largest supine antero-posterior diameter between the xyphoid process and the umbilicus. The participants were asked to inhale and exhale gently, and the arm of the caliper was brought down to touch the abdominal wall without compression.

Percentage body fat was measured with a valid and reliable Omron bioelectric impedance fat monitor, with the participants standing barefoot on the Omron fat estimator. This machine expresses total body fat as a percentage of body fat in kilograms. The FM and FFM were also estimated from participants’ body weight.

### Lung function test

Lung function tests were performed using the Pan African Thoracic Society 2019 guideline (PATS 2019). The test was conducted in an upright sitting position on a chair, and the best curve and the best test were selected following two identical tests that met the acceptability, usability and repeatability criteria for spirometry (PATS 2019). The results were adjusted for race and ethnic correction with a correction factor of 10% (PATS 2019). Daily calibration of the device was conducted using a 3 litres (L) syringe, volume validation at ± 3.0% and linearity flow check at 0.5 L/s – 2.0 L/s for low flow, 4 L/s – 6 L/s for mid flow and 8 L/s – 10 L/s for high flow. All tests were confirmed to have satisfied the recommended ambient conditions of 17° – 35° temperature, 450 millimetre of mercury (mm Hg) – 825 mm Hg of atmospheric pressure and 30% – 75% relative humidity. The following parameters were obtained: forced vital capacity (FVC), forced expiratory volume in 1 s (FEV1), peak expiratory flow rate (PEFR) and FEV1/FVC ratio. All tests were performed by two of the authors who had training in cardiopulmonary conditions and competency training in spirometry and anthropometry. The tests were performed in private cubicles to ensure participants’ privacy.

### Statistical analysis

Descriptive statistics of frequency distribution, mean, standard deviation and percentages were used in summarising the socio-demographic (sex and age) and clinical characteristics (lung function and anthropometry) of the participants. The association amongst selected anthropometric data, age and lung function test parameters was established using Pearson’s product moment correlation tests, whilst *t*-tests were used to establish gender differences in lung function. The statistically significant variables in this bivariate analysis were used as predictor variables in the multiple linear regression analysis. The predictors of lung function were then investigated using multiple linear regression models with subsequent development of regression equations. Age, height, SAD, FM and FFM were the variables entered into the regression model for FVC and FEV1. Age and SAD were variables entered into the regression model for FEV1/FVC ratio, whilst SAD and FFM were entered as variables for PEFR.

### Ethical consideration

Ethical approval to conduct the study was obtained from the Federal Medical Centre Abeokuta Health Research Committee in March 2016 (Ethical Clearance Number: FMCA/16/HREC/038/2016).

## Results

Five hundred apparently healthy employees of two public firms and one private firm were invited to participate, out of whom 444 participated, thus giving a response rate of 88.8%. The mean age of the participants was 37.3 ± 8.2 years (ranging from 22 to 55 years). Fewer than half of the respondents (35.1%, 156) were men. Table 1 shows the summary of the mean, standard deviation and range of anthropometric and lung function parameters measured. Student’s *t*-test analysis revealed a significant difference between male and female participants’ lung function variables (*p*-values for FVC = 0.001, FEV1 = 0.001, PEFR = 0.001) with male participants having significantly higher volumes for FVC and FEV1 and significantly higher flow rate for PEFR (Table 2). However, there was no significant gender difference in the FEV1/FVC ratio (*p* = 0.42).

**TABLE 1:** Summary of mean, standard deviation and range of measured variables (*n* = 444).

Variable	Mean ± SD	Range
Age of participants	37.3 ± 8.25 years	22–55 years
Weight of participants	73.2 kg ± 13.4 kg	51 kg –99 kg
Height of participants	1.66 m ± 0.09 m	1.48 m – 1.92 m
BMI	26.6 kg/m^2^ ± 4.5 kg/m^2^	19.2 kg/m^2^ – 39.66 kg/m^2^
Waist circumference	91.8 cm ± 12.13 cm	70 cm – 113 cm
Hip circumference	106.2 cm ± 9.09 cm	89 cm – 127 cm
Waist-to-hip ratio	0.86 ± 0.06	0.74–0.98
SAD	21.3 cm ± 3.41 cm	15.5 cm – 30 cm
Percentage body fat	31.9% ± 11.19%	7% – 50.7%
FM	23.9 kg ± 10.97 kg	3.79 kg – 50.19 kg
FFM	49.2 kg ± 10.07 kg	35.88 kg – 70.97 kg
FVC	1.69 L ± 0.70 L	0.39 L– 3.07 L
FEV1	1.64 L ± 0.68 L	0.39 L – 3.07 L
FEV1/FVC ratio	0.97 ± 0.04	0.88–1.0
PEFR	4.62 L/s ± 2.5 L/s	0.82 L/s – 12.3 L/s

SD, standard deviation; FEV1, forced expiratory volume in 1 s; FVC, forced vital capacity; BMI, body mass index; SAD, sagittal abdominal diameter; FM, fat mass; FFM, fat-free mass PEFR, peak expiratory flow rate.

**TABLE 2:** Lung function parameters by gender (*n* = 444).

Variable	Men Mean ± SD	Women Mean ± SD	*p*
FVC	2.17 ± 0.63	1.43 ± 0.59	< 0.001
FEV1	2.11 ± 0.60	1.39 ± 0.59	< 0.001
PEFR	6.49 ± 2.89	3.61 ± 1.51	< 0.001
FEV1/FVC ratio	0.97 ± 0.34	0.97 ± 0.35	0.420
*n*	156	288	-

SD, standard deviation; FEV1, forced expiratory volume in 1 s; FVC, forced vital capacity; PEFR, peak expiratory flow rate.

Table 3 shows the correlation between pulmonary function parameters and selected anthropometric variables. Pearson’s product moment correlation revealed that there is a significant but low negative correlation of age and percentage abdominal fat with lung function variables with the exception of FEV1/FVC. Height and weight demonstrated significant low positive correlations with lung function variables except FEV1/FVC. The BMI, WC and HC showed significant weak negative correlations with PEFR and FEV1/FVC ratio, whilst WHR and SAD had low negative correlations with FEV1/FVC. The FM of the participants had a significant negative correlation with lung function variables except FVC, whilst the FFM had a significant moderate positive correlation with FVC and FEV1.

**TABLE 3:** Correlation between lung function parameters and selected anthropometric variables (*n* = 444).

Lung function variable	FVC		FEV1		PEFR		FEV1/FVC ratio	
	*R*	*p*	*R*	*p*	*R*	*p*	*R*	*p*
Age	−0.32	< 0.001	−0.31	< 0.001	−0.35	< 0.001	0.150	0.120
Height	0.50	< 0.001	0.49	< 0.001	0.62	< 0.001	−0.004	0.960
Weight	0.33	< 0.001	0.29	< 0.001	0.17	0.040	−0.360	< 0.001
BMI	0.02	0.810	−0.02	0.850	−0.22	0.010	−0.390	< 0.001
Waist circumference	0.04	0.590	0.02	0.980	−0.16	0.050	−0.390	< 0.001
Hip circumference	0.03	0.720	−0.01	0.950	−0.17	0.040	−0.370	< 0.001
Waist-to-hip ratio	0.04	0.630	0.01	0.930	−0.79	0.340	−0.290	0.001
SAD	0.09	0.280	0.04	0.620	−0.15	0.070	−0.490	< 0.001
Percentage abdominal fat	−0.34	< 0.001	−0.35	< 0.001	−0.51	< 0.001	−0.150	0.070
FM	−0.16	0.060	−0.18	0.030	−0.37	< 0.001	−0.300	< 0.001
FFM	0.61	< 0.001	0.59	< 0.001	0.63	0.630	−0.160	0.060

FEV1, forced expiratory volume in 1 s; FVC, forced vital capacity; PEFR, peak expiratory flow rate; BMI, body mass index; SAD, sagittal abdominal diameter; FM, fat mass; FFM, fat-free mass.

Table 4 shows the main predictors of the pulmonary function parameters. The FVC and FEV1 are predicted by FFM, FM, SAD, height and age of the participants (FVC = 4.28 + 0.072 FFM + 0.012 FM – 0.015 age – 0.055 SAD – 2.834 height; FEV1 = 4.211 + 0.07 FFM – 0.012 age – 0.067 SAD – 2.681 height + 0.013 FM), whilst PEFR is predicted by the FFM and SAD (PEFR = 1.41 + 0.19 FFM – 0.28 SAD). Also, the FEV1/FVC ratio is predicted by SAD and age of the participants (FEV1/FVC ratio = 1.05 – 0.01 SAD + 0.001 age).

**TABLE 4:** Predictors of lung function parameters (*n* = 444).

Variables	Unstandardised beta coefficient	Standard error	Standardised beta coefficient	*t*	*p*	Lower CI	Upper CI	VIF
**FVC**								
Constant	4.280	0.924	-	4.632	0.000	2.464	6.096	-
FFM	0.072	0.007	1.031	9.608	0.000	0.057	0.087	8.885
FM	0.012	0.004	0.195	3.213	0.001	0.005	0.020	2.835
Age	−0.015	0.004	−0.181	−4.276	0.000	−0.023	−0.008	1.385
SAD	−0.055	0.014	−0.265	−3.793	0.000	−0.083	−0.026	3.759
Height	−2.834	0.647	−0.390	−4.201	0.000	−4.159	−1.508	6.642
**FEV1**								
Constant	4.210	0.912	-	4.618	0.000	2.419	6.004	-
FFM	0.070	0.007	1.034	9.489	0.000	0.056	0.085	8.885
FM	0.013	0.004	0.202	3.278	0.001	0.005	0.020	2.835
Age	−0.012	0.004	−0.149	−3.456	0.001	−0.019	−0.005	1.385
SAD	−0.067	0.014	−0.334	−4.707	0.000	−0.095	−0.039	3.759
Height	−2.681	0.666	−0.379	−4.028	0.000	−3.990	−1.373	6.642
**PEFR**								
Constant	1.410	0.577	-	2.444	0.160	−0.580	3.390	-
FFM	0.190	0.009	0.750	21.748	< 0.010	0.160	0.220	1.110
SAD	−0.280	0.025	−0.380	−11.071	< 0.010	−0.370	−0.190	1.110
**FEV1/FVC ratio**								
Constant	1.050	0.009	-	114.625	< 0.010	1.020	1.090	-
SAD	−0.010	0.000	−0.610	−14.871	< 0.010	−0.010	−0.010	1.140
Age	0.001	0.000	0.340	8.137	< 0.010	0.001	0.002	1.140

FEV1, forced expiratory volume in 1 s; FVC, forced vital capacity; VIF, variance inflation factor; CI, confidence interval; SAD, sagittal abdominal diameter; PEFR, peak expiratory flow rate; FM, fat mass; FFM, fat-free mass.

For FVC: *R*^2^ = 0.433 F = 66.8; FVC = 4.28 + 0.072 FFM + 0.012 FM -0.015 age -0.055 SAD -2.834 height.

For FEV1: *R*^2^ = 0.415 F = 62.07; FEV1 = 4.211 + 0.07 FFM -0.012 age -0.067 SAD -2.681 height + 0.013 FM.

For PEFR: *R*^2^ = 0.53 F = 247.09; PEFR = 1.41 + 0.19 FFM -0.28 SAD.

For FEV1/FVC ratio: *R*^2^ = 0.34; F = 115.45; FEV1/FVC ratio = 1.05 – 0.01 SAD + 0.001 age.

## Discussion

Many studies have demonstrated associations between body composition and pulmonary function in both adult and child populations (Fahmy, Khairy & Anwar 2013; Harik-khan, Muller & Wise, 2004; Oloyede et al. 2013; Santana et al. 2001). Our study attempted to document reference lung function values for healthy Nigerian men and women.

Male participants had significantly higher volumes for FVC and FEV1 and significantly higher flow rates for PEFR, which are similar to Glew et al. (2004). Other studies (Neukrich et al. 1988; Oloyede et al. 2013; Soundariya & Neelambikai 2013) also observed higher FVC and FEV1 in men than women. Lung size differences with men having bigger lungs than women may account for this. Men have more FFM, which is synonymous with stronger and more efficient smooth and skeletal muscle. However, the gender of the participants was not significantly associated with the FEV1/FVC ratio, suggesting that this ratio is not gender dependent.

As age increases, the lung volumes and capacities (FVC, FEV1 and PEFR) decrease (Edemeka, Udoma & Ibrahim 2000; Oloyede et al. 2013), as is shown in our results. This may be explained by the loss of the natural elasticity of lung tissue as age increases, resulting in reduced lung volumes and capacities. However, the age of the participants was not correlated with the FEV1/FVC ratio.

Pulmonary function is known to be related to the height and weight of the individual. Our study showed a significant low positive correlation between the height of participants and the lung function variables excluding the FEV1/FVC ratio. Oloyede et al. (2013) and Olanrewaju (1991) showed that pulmonary function parameters correlated positively with height. It can be hypothesised that the lungs increase proportionally with height, which leads to an increase in lung volumes and capacities. The FEV1/FVC ratio differentiates between obstructive and restrictive pulmonary diseases that did not correlate with height.

From our data, it appears that increased lean body mass increases the lung function parameters and increase in percentage body fat decreases most lung function parameters. It is likely that reduced skeletal and respiratory muscle mass in our participants may be responsible (Azad & Zamani 2014). The amount of body fat and a central pattern of fat distribution have been shown to be related to lung function via several mechanisms, such as mechanical effects on the diaphragm (impeding descent into the abdominal cavity) and on the chest wall (changes in compliance and in the work of breathing and elastic recoil) (Lazarus et al. 1997).

The FVC and FEV1 did not significantly correlate with BMI as also seen in Fahmy et al. (2013) and Wannamethee et al. (2005). However, Santana et al. (2001) and Soundariya and Neelambikai (2013) reported a significant negative correlation between BMI and FVC or FEV1. There was a significant weak negative correlation between PEFR, FEV1/FVC ratio and BMI (Atha & Andrew 2014; Lazarus et al. 1997). However, Wannamethee et al. (2005) reported a positive correlation between FEV1/FVC ratio and BMI. The relationship between BMI (a measure of adiposity) and lung function parameters has produced conflicting results from different studies. It is often accepted that an increased adiposity significantly reduces lung function parameters (Fahmy et al. 2013) but these conflicting results can possibly be premised on the fact that BMI is a relative measure of adiposity.

There was no significant correlation between WC of participants and the pulmonary function parameters, which is different from findings from other studies (Fahmy et al. 2013; Soundariya & Neelambikai 2013; Wannamethee et al. 2005; Wehrmeister et al. 2012). These studies demonstrated a significant correlation between WC and pulmonary function parameters but the FEV1/FVC ratio had a low significant negative correlation with the WC. Atha and Andrew (2014) reported a significant negative correlation with the WC and FEV1/FVC ratio. We suggest that increases in WC or HC may be associated with the presence of obstructive or restrictive pulmonary diseases because the FEV1/FVC ratio had a significant low negative correlation with the WC and HC. This is similar to other studies (Fahmy et al. 2013; Wannamethee et al. 2005; Wehrmeister et al. 2012). A high WHR is a strong predictor for the presence of cardiovascular diseases (De Koning et al. 2007; Cao et al. 2018) and it is also significantly associated with reduced FEV1/FVC ratio, that is, the presence of an obstructive pulmonary disease. Perhaps, this may explain why cardiovascular diseases are often accompanied by obstructive lung diseases.

Sagittal abdominal diameter did not significantly correlate with lung function parameters but the FEV1/FVC ratio had a significant low negative correlation with the SAD measurement of participants. Santana et al. (2001) demonstrated a significant negative correlation between FVC, FEV1 and SAD. It appears that SAD is a negative co-correlate of lung function parameters as further analysis revealed that SAD is a negative predictor of lung function parameters.

Sagittal abdominal diameter, FFM and age were important predictors of lung function parameters. The FVC and FEV1 were predicted by FFM and the age of the participants, whilst PEFR is predicted by the FFM and SAD. The FEV1/FVC ratio is predicted by SAD and age. Lazarus et al. (1997) reported that body fat distribution had independent effects on pulmonary function parameters with age having a moderating effect on this pulmonary function variable. These results suggest that aging causes a genuine impact on lung function, leading to a decrease in lung function (Sgariboldi et al. 2016). It has been postulated that as people age, there is progressive decline in lung function signified by obstructive changes, which can be explained by a decrease in elastic lung retractability, a reduction in chest wall compliance and a reduction in the strength of the respiratory muscles (Sgariboldi et al. 2016). Lazarus et al. (1997) also reported that FEV1/FVC ratio is positively predicted by BMI when age is controlled. Our results show that BMI had a significant negative correlation with FEV1/FVC ratio, which is similar to the work of Soundariya and Neelambikai (2013), whilst age and SAD negatively predict FEV1/FVC ratio. Central obesity is considered a systemic inflammatory status that may affect the pulmonary physiology and impact lung function (Wang et al. 2011). This might explain the predictor of SAD on lung function. The BMI is a relative measure of adiposity, but SAD, FFM and FM are more specific measures of adiposity and may accurately predict pulmonary function. It must be stated that FM and FFM are components of body weight that are part of the predictor variables for pulmonary function. Height was reported as a predictor of FEV1 and FVC. The BMI is the ratio of the body weight and height; hence, our result although more specific appear to be similar to Lazarus et al. (1997). Lung function is positively correlated with muscle mass in the trunk or mid-thigh level and negatively correlated with FM in the trunk or central area (Lim et al. 2011). Body composition is related to lung function, and to improve lung function, increases in muscle strength of the trunk and lower extremity with similar reductions of fat in the trunk and upper body may be useful (Lim et al. 2011). The FFM, FM, height, age and SAD are important determinants of lung volumes and key variables for predictive equations of pulmonary function.

Our results may have rehabilitation implications. Including respiratory muscle training amongst this population may improve the respiratory muscles’ strength as aging occurs and thus constitute a preventive strategy against the decline of respiratory muscle strength and pulmonary function (Sgariboldi et al. 2016). People identified as being overweight or obese during rehabilitation should be educated about the implication of excess weight (FM) on lung function. Thus, weight reduction should be added to their treatment management. Although there is a dearth of recent literature on the effect of anthropometry on lung function (which was responsible for old references cited in this study), our study may offer rehabilitation professionals information on this topic.

## Conclusion

Apparently, healthy young Nigerian men have greater lung volumes than women. The FEV1/FVC ratio had low negative correlations with anthropometric indices, and SAD, FFM and age were important predictors of lung function parameters.
